# An Unmanned Aerial Vehicle-Based Gas Sampling System for Analyzing CO_2_ and Atmospheric Particulate Matter in Laboratory

**DOI:** 10.3390/s20041051

**Published:** 2020-02-15

**Authors:** Chaoqun Li, Wenting Han, Manman Peng, Mengfei Zhang, Xiaomin Yao, Wenshuai Liu, Tonghua Wang

**Affiliations:** 1 College of Mechanical and Electronic Engineering, Northwest A&F University, Yangling, Shaanxi 712100, China; chaoqun92@nwafu.edu.cn (C.L.); Tina0808@nwafu.edu.cn (M.P.); 2018050987@nwafu.edu.cn (M.Z.); yaoxiaomin0604@163.com (X.Y.); liuwenshuai@nwafu.edu.cn (W.L.); wangtonghua@nwafu.edu.cn (T.W.); 2 Institute of Soil and Water Conservation, Northwest A&F University, Yangling 712100, China

**Keywords:** UAV-based gas sampling system, gas collection, gas monitoring, particulate matter, propeller airflow

## Abstract

We developed and tested an unmanned aerial vehicle-based gas sampling system (UGSS) for collecting gases and atmospheric particulate matter (PM). The system applies an alternative way of collecting both vertical and horizontal transects of trace gases in order to analyze them in the laboratory. To identify the best position of the UGSS intake port, aerodynamic flow simulations and experimental verifications of propeller airflow were conducted with an unmanned aerial vehicle (UAV) in hover mode. The UGSS will automatically replace the original gas in the system with gas from a target location to avoid the original gas being stored in the air bags. Experimental results show that the UGSS needs 5 s to replace the system’s own original gas using its pump. CO_2_ and PM2.5/10 above the corn field are used as the test species to validate the accuracy of the CO_2_ gas and PM concentrations collected by UGSS. Deming regression analyses showed good agreement between the measurements from the UGSS and the ground sampling station (y = 1.027x – 11.239, Pearson’s correlation coefficient of 0.98 for CO_2_; y = 0.992x + 0.704, Pearson’s correlation coefficient of 0.99 for PM).The UGSS provides a measuring method that actively collects gases and PM for manual analyses in the laboratory.

## 1. Introduction

Gas collecting and detecting is important in many fields, such as toxic leakage gas collection in factories [1,2] and greenhouse gas monitoring in agriculture [3,4,5], forestry [6,7] or animal husbandry [8,9,10]. At present, there is a lot of research available on gas collection and analysis. Schuck analyzed the main greenhouse gases, CO_2_, CH_4_, N_2_O, and SF_6_, collected by air sample equipment from the Civil Aircraft for the Regular Investigation of the Atmosphere Based on an Instrument Container (CARIBIC) instrument package [11]. Van developed a mini-denuder sampling device for direct desorption in an inlet of a gas chromatograph for the analyses of airborne gaseous-phase pollutants and airborne particulate-phase pollutants [12]. Stavert measured greenhouse gas emissions using two new tall towers [13]. The towers were equipped with a combination of a cavity ring-down spectrometer, gas chromatograph instrumentation and automated gas sampling systems.

With the development of unmanned aerial vehicle (UAV) technology, UAVs have been deployed for gas monitoring and subsequent research. UAVs equipped with multiple gas sensors [14,15] can be used to directly measure gas emissions from industrial stacks or ground vehicles where it is too difficult or dangerous to use piloted aircraft [16] or to build ground-level stations [17]. Furthermore, more specialized designs have been developed and implemented for specific gas and particulate matter (PM) monitoring. A gas sensor system for monitoring pollutant gases in the low troposphere using a small UAV was created to identify, quantify, and assess the flux exchange of pollutant gases between the Earth’s surface and atmosphere [18]. Peng et al. monitored the vertical distribution patterns of PM2.5 concentrations based on UAV technology in Hangzhou, China [19]. Rudiger et al. measured volcanic gases using electrochemical, optical, gas sensor, and manual sampling devices mounted on a UAV [20]. An active UAV-based AirCore system was designed to measure the atmospheric mole fractions of CO_2_, CH_4_, and CO in 2018 [21]. 

While UAVs are actively deployed for gas monitoring, deficiencies in current UAV-based monitoring equipment still need to be addressed. The UAV propellers can cause airflow disturbance, which will change the concentration of the original gas and PM in conditions without UAV airflow [22,23,24]. Roldan et al. found that gas measurements with moving and stationary rotors are different and the average relative errors for temperature, humidity, and CO_2_ concentration are 3.71%, 1.65%, and 3.84%, respectively [25]. To reduce this effect caused by propeller airflow, many authors have analyzed the airflow to determine the optimum installation positions of monitoring sensors [26,27]. Temperature, humidity, and CO_2_ sensors have been distributed and placed after studying the quadrotor aerodynamics and influence of airflow on the rotors of a UAV [22]. More complex analyses include the 3D visualization of different airspeed regions produced by the IRIS+ 3DR UAV propellers [28] to identify the best positions for the sensor probes on the side of the UAV.

Although many researchers have conducted UAV-based gas monitoring [29,30], the limitations of UAV airflow and UAV airborne sensors need to be addressed. Current airflow analysis does not adequately characterize the airflow caused by propeller or UAV flight speed during gas monitoring or when the sampling system is active [28]. Moreover, the accuracy of airborne sensors often cannot meet the accuracy requirements and gas samples should be analyzed in the laboratory using more accurate measuring equipment, such as a gas chromatograph.

To improve measuring accuracy, a UAV-based gas sampling system (UGSS) for collecting gases and PM when the UAV is in hover mode was to be developed. Experiments were carried out in the process of the system design and our objectives were threefold: (1) to identify the best position of the UGSS intake port, (2) to identify how long it takes the UGSS to replace the original gas with gas from the target location using its pump to avoid the original gas being stored in the air bags and (3) to validate the accuracy of gas and PM concentration collection by the UGSS.

## 2. Materials and Methods

### 2.1. The Unmanned Aerial Vehicle-Based Gas Sampling System

**Figure 1:**
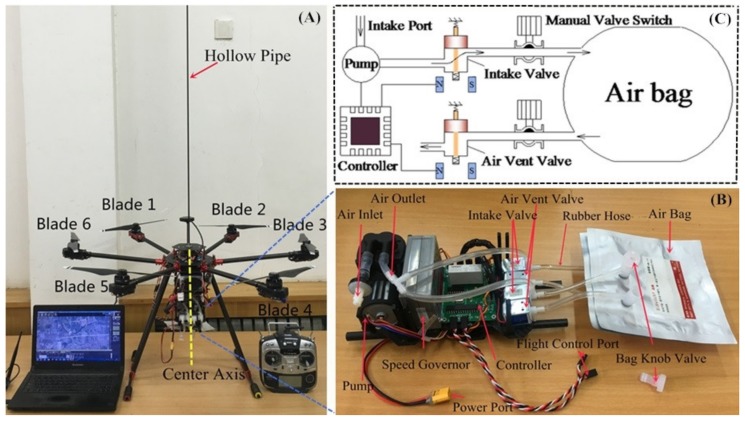
(**A**) The yellow dotted line is the center axis and air intake hollow pipe (AIHP) was mounted on the center axis. Six blades are shown on the picture. (**B**) An image of the gas sampling equipment (GSE). (**C**) The gas collection schematic and the state of each part during the original gas was replaced by the gas from the target location using the pump.

The UGSS (Figure 1A) includes a computer, UAV, gas sampling equipment (GSE), remote controller, and air intake hollow pipe (AIHP) (carbon fiber pipe, internal diameter: 4 mm, length: 120 cm). The computer is used to plan the routes and monitor the equipment. The remote controller is used for manual control of the UAV and GES. AIHP was used to collect the gas in the location, undisturbed by UAV airflow turbulence. The UGSS has two modes to collect gases and PM: (1) manual, via remote control, and (2) automatic, using a preplanned UAV route and GPS information designed in the ground control software Mission Planner (open-source UAV ground station, created by Michael Oborne in 2010).

#### 2.1.1. The Unmanned Aerial Vehicle

The UAV used in this study is a hex copter with a 65.4 cm center frame diameter and 55.4 cm height, equipped with DJI1345-s propellers (SZ DJI Technology Co., Ltd. Shenzhen, China) and landing gear. The aircraft weighs 4.9 kg and has a 10 kg maximum acceptable gross takeoff weight. The UAV is equipped with a 16,000 mAh battery and has a maximum flight time of approximately 15 min when the GSE is mounted. An in-field test of data transmission capability showed no loss in signal strength at a distance of 1025 m. The UAV is equipped with the PIXHAWK Flight Controller from 3D Robotics (Berkeley, CA, USA) and the flight controller also uses a multi-rotor stabilization controller for navigation, flight controls, and autopilot with an inertial measurement unit (IMU) and GPS. The UAV can fly preprogrammed flight paths or be manually controlled by the pilot. The pilot can initiate an automatic or manual landing. The UAV has a return-to-home function when the batteries reach a preset charge threshold. An array of automatic response actions cover all event contingencies, such as loss of command and control signal, loss of GPS signal, propeller motor failure, or low voltage. The flight controller has six auxiliary channels that can output level or pulse width modulation (PWM) signals at specific times and locations set by the user when flying a planned UAV route using Mission Planner.

#### 2.1.2. Gas Sampling Equipment 

The GSE (Figure 1B) mounted on the UAV was established to collect gases and PM by manual remote control or automatic gas acquisition, using a preplanned UAV route. The designed GSE weighs 0.6 kg and consists of a controller, a micro pump, a micro pump speed governor, four two-position two-way electromagnetic valves, rubber hoses (internal diameter: 4 mm, length: 325 mm each bag), and two air bags. The model, manufacturer, dimensions, number and performance of the different parts used for the development of the gas sampling equipment are shown in Table 1. When the controller receives the relay signal from the remote controller or flight controller, the GSE is turned on for sampling. The working process is explained as shown in Figure 1C. First, the pump, the intake valve and the air vent valve were turned on. Concurrently, the original gas in the hoses and air bag was replaced by the gas from the target location using the pump. After the original gas was replaced, the air vent valve closed and the bag began to store gas. Finally, when gas collection was complete, the pump turned off and the intake valve was closed. After one gas collection was completed, the UAV flew to the next target location to collect gas using another air bag or returned to the home point. After the UAV brought back the sampling gas, we sealed the air bags using the manual valve switch and took them away for measurement. 

### 2.2. Rotation Speed and Propeller Gas Flow Measurement

**Figure 2:**
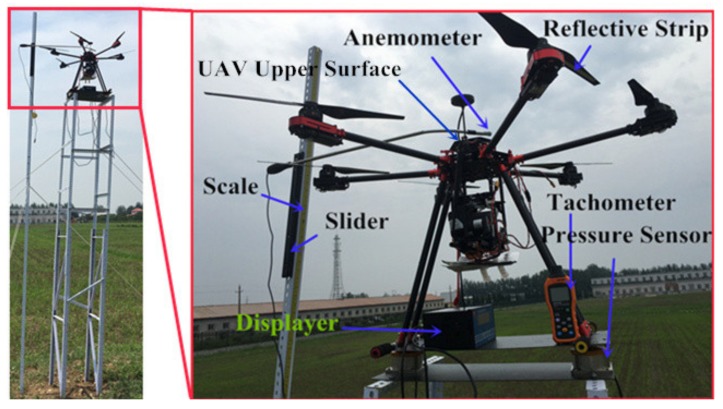
Experimental configuration to measure propeller gas flow and rotation speed of the UAV. Left: overall structure of platform; Right: details of the platform.

The UAV should be in hover mode when the UGSS collects the gas. To determine the position of the intake port with little propeller air turbulence, a platform was set up to measure the gas flow and rotation speed of the propellers when the UAV is hovering. Figure 2 shows the platform for the experiment. The UAV is firmly attached to the pressure sensors, fixed on a bracket. The bracket is 4 m high to ensure minimal propeller airflow interference from the ground. A supporting rod with a scale and sliding guide was fixed around the bracket. The air speed at different positions was measured by adjusting the position of the anemometer fixed on the sliding guide. The lift force provided by the propellers of the UAV was controlled by remote control. When the UAV lift force was equal to its gravity, the propeller revolution speed and air velocity on the center axis above the UAV upper surface is measured. The UAV airflow was simulated using the mean value of propeller revolution in the fluid simulation software FLUENT18.0 (ANSYS, Inc. Canonsburg, PA, USA). The air speed was measured in a calm environment and the velocity was measured three times. The measurement began at the UAV upper surface and, then was conducted 2 cm above the previous height for each measurement that followed. Most of the gas resistance to shear (i.e., viscosity) is the result of the transfer of molecular momentum. Generally speaking, the viscosity of a fluid increases only slightly with pressure. The higher the temperature, the larger the transfer of molecular momentum because molecules move faster and the viscosity of a gas increases with rising temperature [31]. At the same speed of the propeller, the higher the temperature, the smaller the influence range of propeller airflow. Therefore, the air inlet position selected in low temperature environments is also applicable when the temperature increases. In order to adapt to most temperature conditions when collecting gas, 0 ℃ was selected for the temperature conditions of the simulation and experiment. The anemometer (model AR866A, XiMa Co., Ltd. Dongguan, China) measurement error was 0.1 m/s + 5% of reading (0–2 m/s) and 0.3 m/s + 5% of reading (2–15 m/s). The revolution counter (model MS6208B, HuaYi Co., Ltd. Dongguan, China) had a measurement error of ± (0.04% + 2) and a measuring range of 1000–9999 RPM. 

### 2.3. Original Gas Discharge Checking System

**Figure 3:**
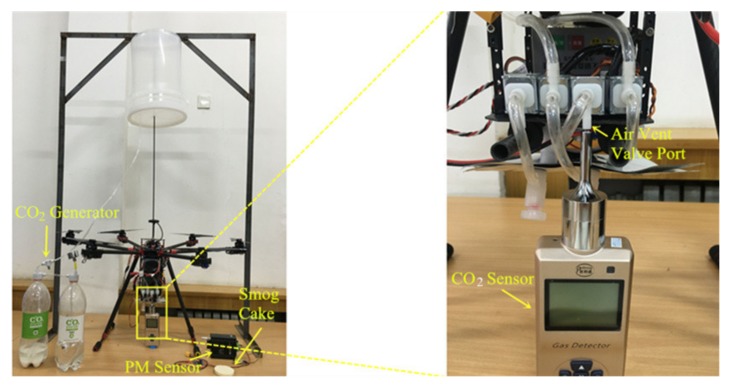
Experimental configuration to identify how long it takes to replace the original gas in unmanned aerial vehicle-based gas sampling system (UGSS) with gas from the target location using its pump to avoid the original gas being stored in air bags.

Before gas collecting, the original gas in the AIHP, rubber hoses, and airbag should be replaced. To identify how long it takes to replace the original gas with the gas from the target location using the pump (flow rate 0.4 L/min), a measurement platform was established (Figure 3). Sodium bicarbonate and 2-hydroxy-1,2,3-propanetricarboxylic acid (Xinfan Biotechnology Co., Ltd. Shanghai, China) were used as a CO_2_ generator. The principle is as follows:

C₆H₈O₇ + 3 NaHCO₃ = C_6_H_5_O_7_Na_3_ + 3H_2_O + 3CO_2_

When the barometer value is greater than 1.5 kgf/cm^2^, open the valve of the CO_2_ generator, and pass the CO_2_ into the container through the rubber hose (internal diameter: 4 mm, length: 710 mm) for 30s. Smog cake (Xincheng Co., Ltd. Tianjin, China), i.e., mainly potassium nitrate, alpha-D-Glucopyranosyl beta-D-fructofuranoside, and ammonium chloride are often used in fire drills and can produce PM when ignited. In the experiment, take 100 gram smog cake, ignite it and put it in the container to make PM. The purity grade of the reagent used in the experiment was laboratory purity. The container has two holes: one used to stabilize the air pressure and the other connected to the UGSS inlet port or sensor inlet port. The CO_2_ and PM concentration can be kept constant for a long time, thus providing a stable environment for the experiment. We used the following experiments to determine the time required for original gas discharge. In the port of the air vent valve, either a PM sensor or a CO_2_ concentration sensor was placed to measure the gas concentration. At the beginning, the gas measured by the sensor is the original gas in the system. When the original gas in the system is discharged, the air vent valve port begins to discharge the gas in the container. Concentration data is recorded once per second. Since the concentration of PM or CO_2_ in the container is much higher than that of the original gas in the system, the time for original gas discharging can be determined by analyzing the change in concentration value. Due to the delay characteristic of the sensor, it is necessary to determine the delay time of the sensor during the experiment. The experiment was carried out three times and average value was calculated every second. The CO_2_ gas concentration sensor Pulitong (model XLA-BX, Guotai Fire Group Co., Ltd. Nanjing, China) had a measuring range of 0–5000 ppm and error of 30 ppm. The pump suction Yongteng PM sensor (model YT-PM2510, Yongteng Electronics Co., Ltd. Huizhou, China) can provide stable airflow for monitoring. This sensor had a measurement error of less than 10 μg/m^3^ (0 to 100 μg/m^3^) and 10% of full scale (100 to 2000 μg/m^3^). Data can be recorded from 1 s to 1 h intervals depending on experimental needs. 

### 2.4. Ground Gas Sampling Station and Flight Design

**Figure 4:**
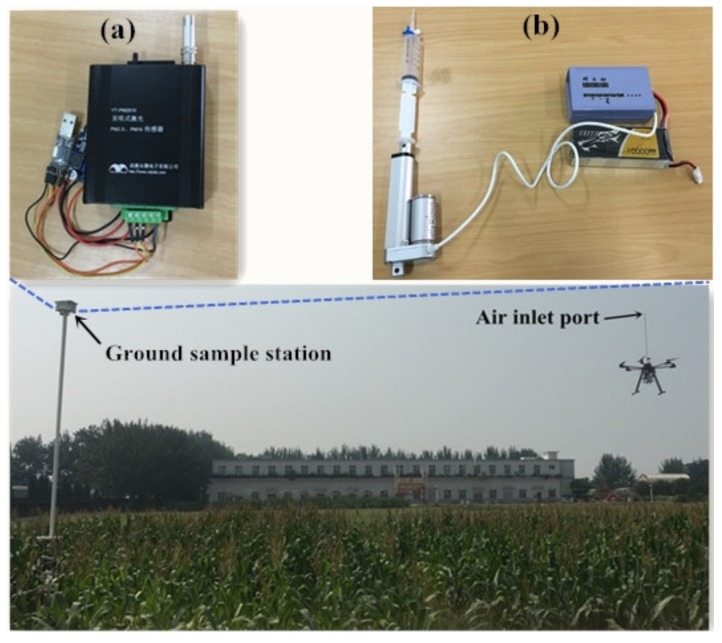
Image showing flight operation experiments on Septeimber 3, 2019. (**a**) Particulate matter sensor in ground sample station. (**b**) Polypropylene syringes equipped with an automated lifter drawing bar in ground sample station.

An experiment was designed to compare the data collected from UGSS with data from the ground gas sampling station (GSS) (Figure 4). The GSS was 7 m from the ground. The GSS contains polypropylene syringes equipped with an automated lifter drawing bar (Figure 4b) and the Yongteng PM sensor (Figure 4a). It is a common measurement method to collect gas with a syringe and analyze it using a chromatograph [32,33]. A polypropylene syringe was used to collect gas at the GSS at the time when the UGSS collected the gas. PM sensors were used to measure and record the PM value at the GSS at the time when the UGSS collected gas. The value from the GSS is taken as the true value rather than the UGSS data. The measurement was conducted on September 3, 2019, during the corn milk stage at the Northwest A&F University, Yangling, China. On the day of the experiment, there was a light wind of force two on the Beaufort scale and the wind direction was southwesterly. All flights cruised along the same route to collect gas 15 m from the ground station location (34.2985328N 108.0686050E) in the corn field. When UGSS collected gas, the UGSS air inlet port and the GSE were the same height from the ground. The UAV takes off from the home point, flying horizontally at an altitude of 5 meters, then vertical climbs to the gas sampling point and hovers for gas collection. After gas collection, the UAV automatically returns to the home point. Gas collection was conducted once every hour from 7:00 to 18:00. A total of 12 gas samples were collected. The collected gas from the UGSS and the GSS were measured using gas chromatograph for CO_2_ (GC-2010PLUS, Shimadzu, Japan) and sensor Yongteng for PM. In this research, the same PM sensor was used for the determination of PM in the lab, for PM measurement at the ground gas sampling station and for the evaluation of the time required to discharge the residual gas in the UGSS. CO_2_ concentration was analyzed using a gas chromatograph with a thermal conductivity detector (TCD). High-pure nitrogen with a flow rate of 30 mL min^−1^ was used as a carrier gas. The temperatures of the detector, column box, and injector port were 120, 50, and 100 °C, respectively.

## 3. Results and Discussion

### 3.1. Unmanned Aerial Vehicle Air Turbulence in Hover Mode and Position of the Unmanned Aerial Vehicle-Based Gas Sampling System Intake Port

When the UAV is in hover mode, the speed of each propeller on the UAV adjusts based on the flight attitude. Although the UAV was placed horizontally, the propeller rotation speeds are slightly different because of installation errors. As shown in Table 2, when the UAV lift force was equal to its gravity, the rotation mean value for all six blades was approximately 5650.2 revolutions per minute (rpm). 

**Figure 5:**
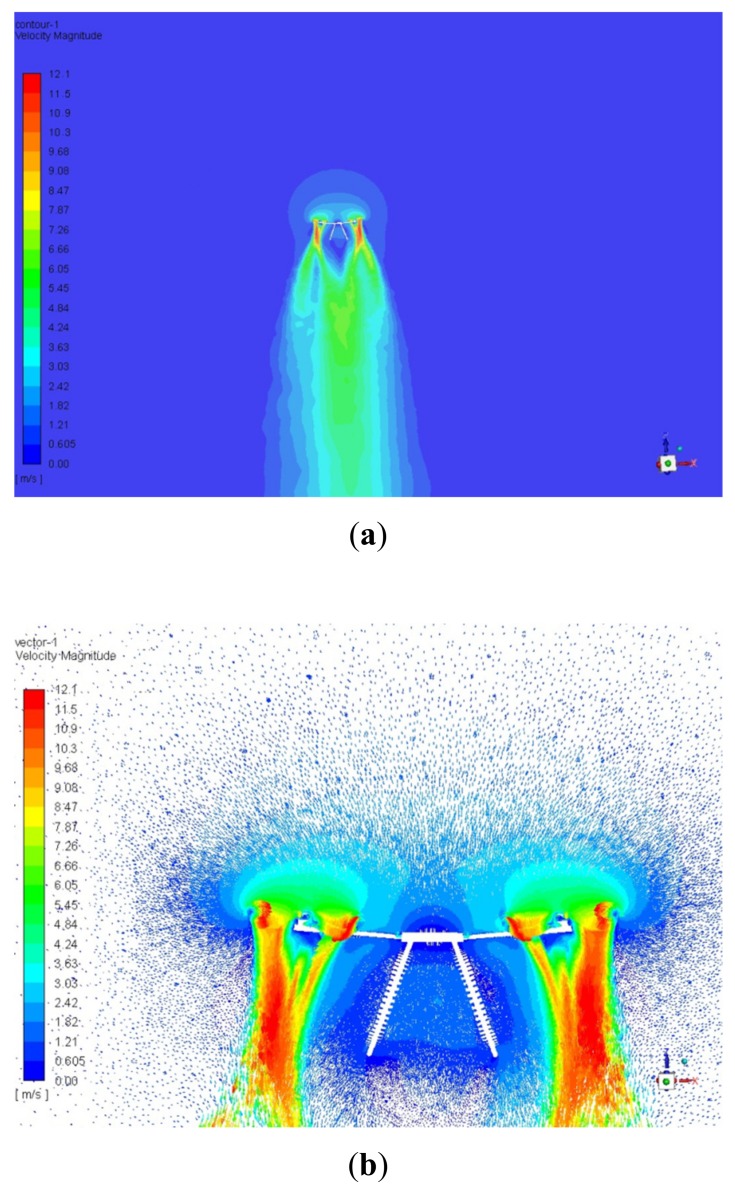
Results from the airflow simulations, (**a**) velocity magnitude and (**b**) velocity vector.

The UAV airflow was simulated with this mean value in the fluid simulation software FLUENT 18.0 (ANSYS, Inc. Canonsburg, PA, USA). As shown in Figure 5a, the maximum velocity magnitude of the air flow was 12.1 m/s under the blades. The air flow velocity declined gradually beneath the UAV. The air velocity above the UAV was smaller compared to the velocity under the UAV and declined with the distance from the UAV upper surface. Above the surface, the simulation results indicate four airspeed layers in the center axis, as shown in Table 3. When the distance reaches 120 cm, the air speed starts to become 0 m/s. From the velocity vector (Figure 5b) we know that the direction of the airflow on the UAV center axis goes from up to down.

**Figure 6:**
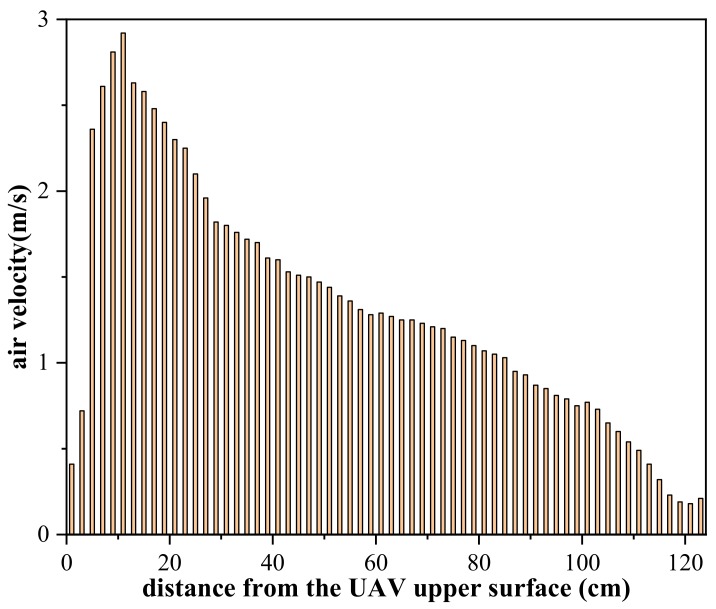
Air velocity of UAV center axis above the UAV upper surface.

The air velocity average values were measured in Figure 2, as shown in Figure 6. The velocity magnitudes are very small, from 1 to 3 cm above the upper surface, because gas flow is blocked by the UAV upper surface. From 3 cm to 5 cm, the airflow speed increases quickly from 0.72 m/s to 2.36 m/s. Air velocity gradually increases from 5 to 11 cm and declines from 11 to 120 cm. When the distance reaches about 120 cm, the air velocity starts to become calm (0–0.2 m/s according to the Beaufort scale). Both the simulation and experiment results show that the air velocity increases rapidly and then decreases slowly, but there are some differences in the numerical value: the experimental maximum air velocity is 2.85 m/s, while the simulation is only 2.42 m/s; at 120 cm, the simulated airflow value is 0.605 m/s and the experimental value is about 0.2 m/s. The possible reasons for this difference are as follows: (1) The simulated environment is windless (0 ℃), but it is not easy to ensure the experimental environment temperature constant remains at 0 ℃, and small natural winds can also cause numerical differences. (2) There is no air flow blockage in the simulation environment, however, both the frame and the ground will affect the propeller air flow in the experiment. (3) The measured air speed is the sum speed of the natural flow and propeller air flow in a vertical direction. With the increase in the distance from the UAV’s upper surface, the influence of the propellers on the total flow direction decreases and the influence of natural air flow on the total flow direction increases. Since the air velocity is small after 120 cm, the gas inlet position was set in 120 cm above the UAV upper surface.

### 3.2. Time Required to Discharge Residual Gas in Unmanned Aerial Vehicle-Based Gas Sampling System

**Figure 7:**
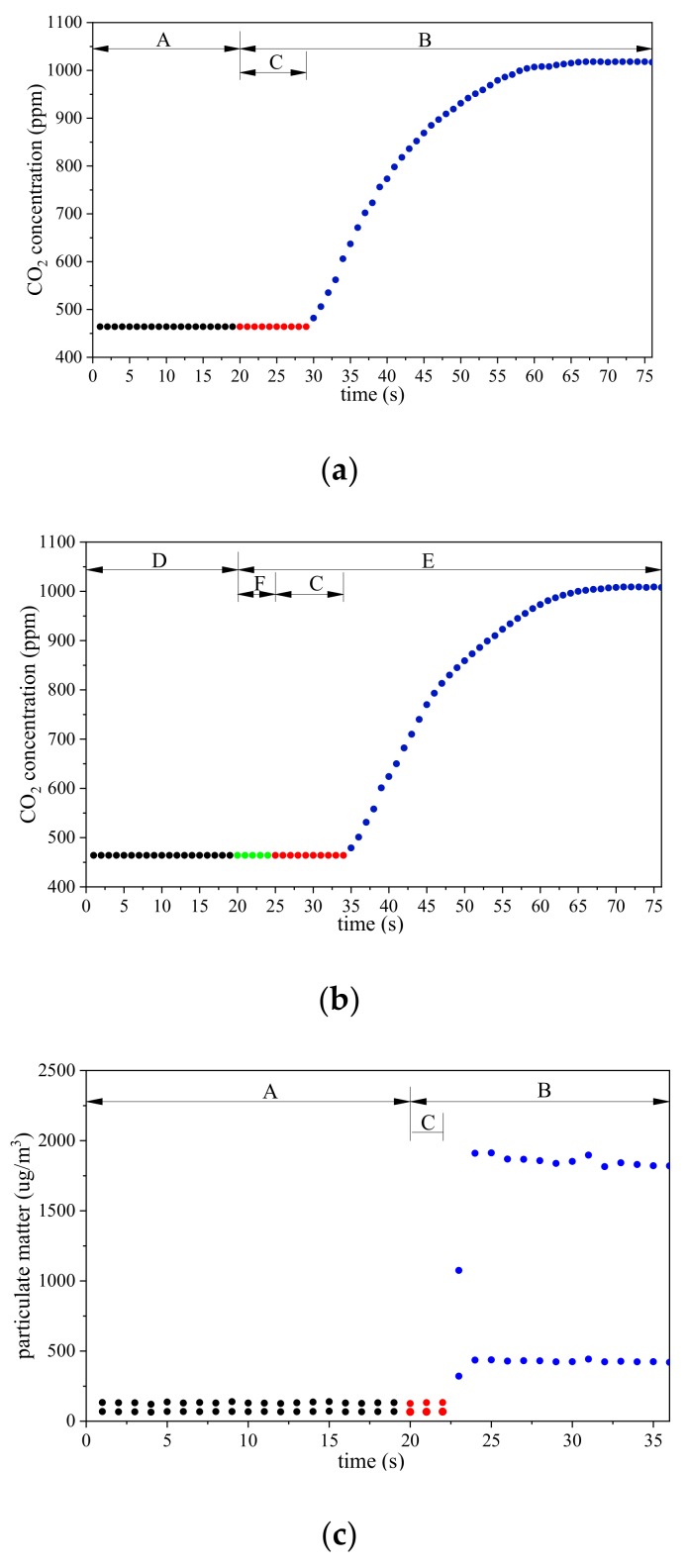

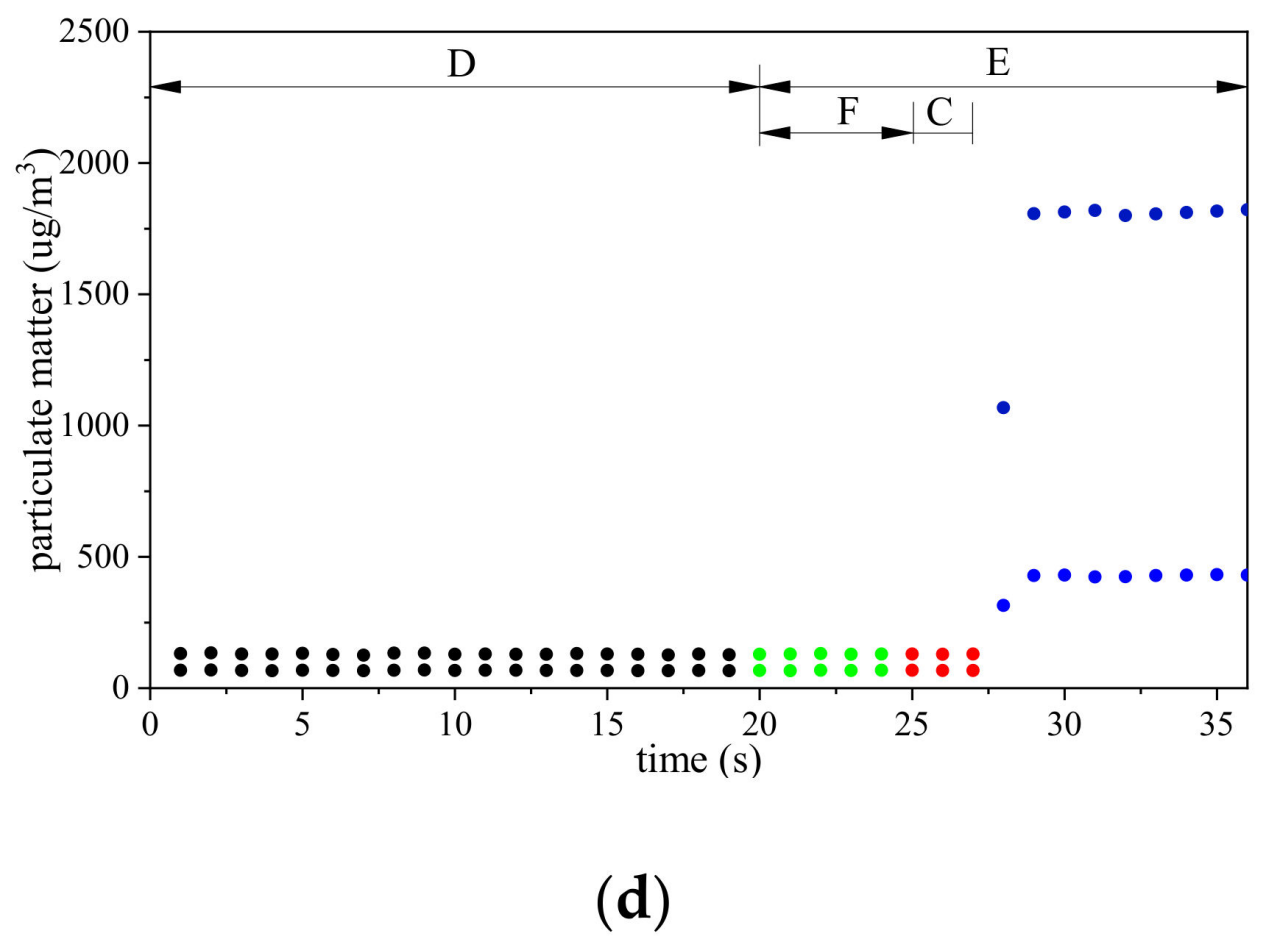
(**a**) CO_2_ concentration values in the experimental of CO_2_ sensor delay. (**b**) CO_2_ concentration values in the experimental of time required to discharge residual CO_2_ gas in UGSS. (**c**) Particulate matter (PM) PM2.5/PM10 concentration values in the experimental of CO_2_ sensor delay. (**d**) PM2.5/10 concentration values in the experiment of time required to discharge residual CO_2_ gas in UGSS.

Figure 7a shows the experimental results of the CO_2_ sensor delay measurement. Period A was the CO_2_ concentration change in the natural environment (464 ppm). Period B was the CO_2_ concentration change when the sensor’s gas inlet was inserted into the container. Period C was the sensor delay time and, in this period, the CO_2_ concentration value remained at 464 ppm. After Period C, the sensor values began to increase, initially quickly and then slowly. The value remained at approximately 1018 ppm after the 31 s sensor value growth. The experiment confirmed that the CO_2_ sensor delay time was 10 s.

Figure 7b shows the experimental results of the time required to discharge the residual CO_2_ gas in UGSS. Period D was the CO_2_ concentration change in the natural environment in the air vent valve port of the GSE (464 ppm). Period E was the CO_2_ concentration change in the same position when the air intake port of the UGSS was inserted into the CO_2_ container. From the experimental results of the CO_2_ sensor delay measurement, we know that Period C was 10s. Therefore, Period F was for discharging the residual gas from the UGSS. After Period C, the sensor values began to increase, initially quickly and then slowly. The value approximately remained at 1008 ppm after 31 s of sensor value growth.

Figure 7c shows the experimental results of the PM sensor delay measurement. Figure 7d shows the experimental results of the time required to discharge the residual PM gas in the UGSS. The meaning of the letters in Figure 7c,d is the same as that of Figure 7a,b, except that the experimental object is replaced by the PM. The experiment confirmed that the PM2.5/10 sensor delay was 2 s. The time required to discharge the residual PM gas is the same as that of CO_2_.

The experiment confirmed that the UGSS needs an additional 5s to discharge the residual PM and CO_2_ gas in the AIHP, rubber hose and airbag using the internal pump.

### 3.3. Accuracy Validation of the Unmanned Aerial Vehicle-Based Gas Sampling System

#### 3.3.1. Accuracy Verification of CO_2_ Concentration Collected Using Unmanned Aerial Vehicle-Based Gas Sampling System

**Figure 8:**
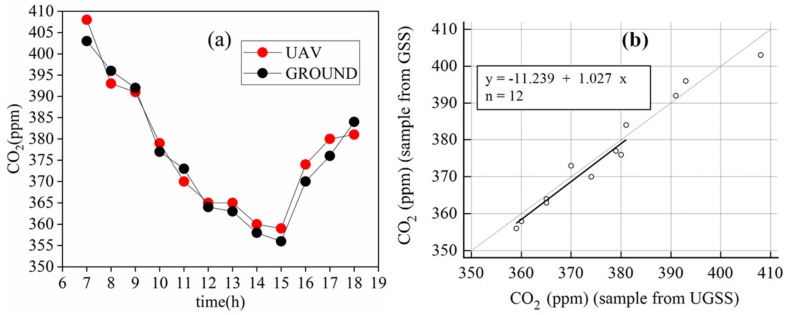
In graph (**a**): the PM2.5/10 concentrations collected by the UGSS and concentrations recorded by PM sensor at ground gas sampling station (GSS). In graph (**b**): Deming regression analysis of the CO_2_ concentration sample from UGSS vs. GSS.

Figure 8a shows the CO_2_ concentrations collected by the UGSS and polypropylene syringes from 7:00 to 18:00. Figure 8b shows the correlation between the CO_2_ concentrations collected by the UGSS and polypropylene syringes. 

As shown in Figure 8a, the measurements from the UGSS and GSS were in very good agreement from 7:00 to 18:00. The highest value was observed at 7:00, which may be due to the overnight respiration of the crops. Crops can absorb a lot of CO_2_ through photosynthesis, and the concentration of carbon dioxide decreases continuously from 7:00 to 15:00. Then the concentration of CO_2_ begins to increase until 18:00 as the light weakens. There is a strong correlation between the CO_2_ concentration measurements from the UGSS and polypropylene syringes at the GSS. The two values measured per hour were used to form a coordinate point. Deming regression analysis (Figure 8b) of the sample from the UGSS vs. the GSS based on these 12 CO_2_ concentration samples yielded a slope of 1.027 (95% CI, 0.797–1.267), an intercept of -11.239 ppm (95% CI, −100.047–77.569) and a Pearson’s correlation coefficient of 0.98.

#### 3.3.2. Accuracy Verification of Particulate Matter Concentration Collected Using the Unmanned Aerial Vehicle-Based Gas Sampling System

**Figure 9:**
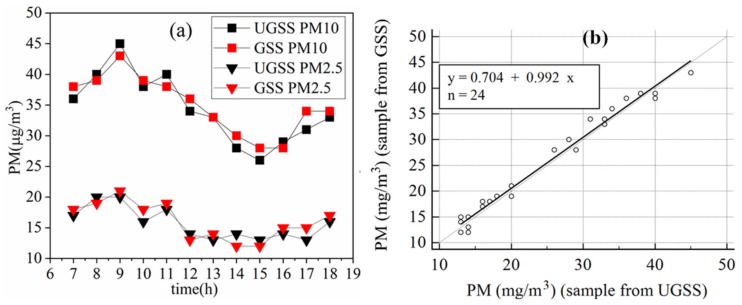
In graph (**a**): the PM2.5/10 concentrations collected by the UGSS and concentrations recorded by PM sensor at GSS. In graph (**b**): Deming regression analysis of the PM2.5/PM10 concentration sample from UGSS vs. GSS.

Figure 9a shows the PM2.5/10 concentrations collected by the UGSS and the concentrations recorded by the PM sensor from 6:00 to 19:00. Figure 9b shows the correlation between the PM2.5/10 concentrations collected by the UGSS and those recorded by the PM sensor at the GSS. 

As shown in Figure 9a, the measurements from the UGSS and GSS were in very good agreement from 7:00 to 18:00. Before the highest value was obtained at 9:00, the PM concentration increased between 6:00 and 9:00 and then decreased between 9:00 and 15:00. After reaching its lowest value at 15:00, it began to increase until 18:00. The concentration of PM increased twice, which may be related to the vehicle’s travel during its commuting time. There is a strong correlation between the PM2.5/PM10 concentration measurements from the UGSS and the GSS. The values measured every hour using the two methods were used to form a coordinate point. Deming regression analysis (Figure 9b) of the sample from the UGSS vs. that of the GSS, based on these 24 PM2.5/PM10 concentration samples, yielded a slope of 0.992 (95% CI, 0.915–1.069), an intercept of 0.704 µg/m^3^ (95% CI, −1.16–2.567) and a Pearson’s correlation coefficient of 0.99.

## 4. Conclusions

In this study, a UGSS was developed and tested in the laboratory and in experimental flights. The laboratory test results for propeller air turbulence have shown that the airflow speed above the UAV experienced three processes: rapid increase, slow increase and slow reduction. When the distance from the UAV upper surface reaches about 120 cm, the air velocity starts to become calm and the UGSS air intake port was installed in this location. The UGSS requires 5s to discharge the system’s residual gas and PM using the internal pump.

Through the analysis of the data from the UGSS and the GSS, we know that the UGSS can obtain accurate CO_2_ and PM concentrations. The UGSS can collect both vertical and horizontal transects of trace gases and provide a measuring method that actively collects gases and PM for manual analyses in the laboratory.

## Figures and Tables

**Table 1:** Introduction to main parts of gas sampling equipment.

Part	Model	Dimensions	Performance	Manufacturer
Micro pump	FNY6003	92×83×48 (mm)	maximum flow: 3L/min	Xin Wei Cheng Co., Ltd. China
Speed governor	KS-1	80×45×25 (mm)	Flow regulation range: 0-3 L/min	Xin Wei Cheng Co., Ltd. China
Electromagnetic valves	PYF3	30×20×12 (mm)	2-position 2-way	Pin Cheng Co., Ltd. China
Air bag	E-Switch	150×150 (mm)	2 valves, 0.5 (L)	Sunrise Instrument Co.,Ltd. China

**Table 2:** Rotation values for each blade when the lift force was equal to the system’s gravity.

Blade Number	Rotation Direction	Rotation Speed (rpm)
Blade 1	Clockwise	5649
Blade 2	Counter-clockwise	5647
Blade 3	Clockwise	5651
Blade 4	Counter-clockwise	5654
Blade 5	Clockwise	5653
Blade 6	Counter-clockwise	5647

**Table 3:** Air speed range on the central axis above the unmanned aerial vehicle (UAV) upper surface.

Location Range on the Central Axis above UAV Upper Surface (cm)	Air Speed Range (m/s)
0–3	0–0.605
3–60	1.82–2.42
60–70	1.21–1.82
70–120	0.605–1.21
